# Patient specific Polymethyl methacrylate customised cranioplasty using 3D printed silicone moulds: Technical note

**DOI:** 10.1002/rcs.2353

**Published:** 2021-11-24

**Authors:** Alba Scerrati, Francesco Travaglini, Clarissa Ann Elisabeth Gelmi, Andrea Lombardo, Pasquale De Bonis, Michele Alessandro Cavallo, Paolo Zamboni

**Affiliations:** ^1^ Department of Translational Medicine University of Ferrara Ferrara Italy; ^2^ Department of Neurosurgery Sant'Anna University Hospital of Ferrara Ferrara Italy; ^3^ 3D Bioprinting Laboratory University of Ferrara Ferrara Italy; ^4^ Hub Center for Venous and Lymphatic Diseases Regione Emilia‐Romagna Sant'Anna University Hospital of Ferrara Ferrara Italy

**Keywords:** 3D‐printing, craniofacial, cranioplasty, custom‐made, modelling, neurosurgical trauma, PMMA, prosthetics, reconstruction

## Abstract

**Introduction:**

Cranioplasty after decompressive craniectomy can be performed with several techniques and materials. With the common use of 3D printing, custom cranioplasty can be produced at affordable cost. Aim of this technical note is to describe our technique for producing patient specific Polymethyl methacrylate (PMMA) cranioplasty using 3D printed silicone moulds.

**Materials and Methods:**

We enrolled seven patients from January 2020 to June 2021 who required surgery for cranioplasty. The 3D printing was used to produce silicone moulds for defining the exact shape of the PMMA cranioplasty, according to the CT scan of the patient.

**Results:**

We performed seven procedures. The mean time of the surgery was 80 min. All cranioplasties perfectly matched the patient specific anatomy. No complications occurred.

**Conclusions:**

Using 3D printed patient specific silicone moulds and PMMA resulted to be effective, with affordable costs and ensuring a good cosmetic result.

## 1 INTRODUCTION

Cranioplasty is a common procedure performed to repair cranial defects. When autologous bone is not available, a reconstruction with alloplastic material is required.

Customised cranial implants with PEEK or titanium are often used, achieving good aesthetic results. Indeed, their cost still remains high.^1^

Polymethyl methacrylate (PMMA) is often used to perform cranioplasty. It is biocompatible, easily shapeable and low‐cost. However, its free‐hand modelling often results in a poor cosmetic result.^2^

With advances of 3D printing technologies, possibilities to produce low‐cost patient specific PMMA cranioplasty emerged, in particular exploiting a mould technique.^1^, ^2^, ^3^, ^4^, ^5^, ^6^, ^7^

We would like to describe our procedure for performing a low‐cost patient specific PMMA customised cranioplasty using 3D printed silicone moulds.

## 2 MATERIALS AND METHODS

### 2.1 Patients

This is a prospective observational study. We enrolled all patients who underwent a decompressive craniotomy requiring surgery for cranioplasty from January 2020 to June 2021.

Full ethical approval was obtained from the Research Ethics Committee (ref: 15/EM/01898) before the study commenced, ethical guidelines followed, and informed consent sought from all patients.

### 2.2 3D printing technique

The technique was developed by the department of Neurosurgery and the 3D bioprinting Laboratory at University of Ferrara.

Pre and post decompressive craniotomy high resolution head CT scan DICOM files were collected. These were converted in a 3D model and subsequently in a .stl file using the InVesalius 3.1, VxElements, Rhinoceros, Meshmixer, Simplify3D software. The .stl model was elaborated with the software in order to reconstruct the defect area. The pre‐decompressive craniotomy was used as reference to elaborate the 3D model of the skull defect. When the original bone was not available from previous CT scans, we usually applied the ‘mirror technique’, using the healthy contralateral side to obtain the cranioplastic shape. The reverse engineered .stl model of the defect was used to create the 3D patient‐specific mould composed by two pieces which fit inside each other (see Supporting Information S1, Video 1). A central 1 cm hole was planned in order to pour the PMMA in the mould during the surgery.

The mould .stl was transmitted to a company specialised in the creation of 3D printed silicone medical devices to create the 3D mould using the WASP 4070 Industrial printer. Printer parameters were as follows: nozzle temperature: 220°C; bed temperature: 55°C; mean time of printing: 9 h; default printing speed 45 mm/s; *X*/*Y*‐axis movement speed 150 mm/s; *Z*‐axis movement speed 150 mm/s.

Once the two pieces of silicone mould were ready, they were sterilised in autoclave for 1 h at the highest temperature of 134°C and 2.7 bar and packed for the intraoperative use.

The volume of the required PMMA to obtain a 3‐mm‐thick cranioplasty was calculated using millimetered becher and water and subtraction to get an approximation of the volume.

### 2.3 Surgery

Previous surgical incision was re‐opened and the skin flap was dissected from the underlying dural plane, isolating the temporal muscle. All the borders of the previous craniotomy were clearly exposed.

**FIGURE 1:**
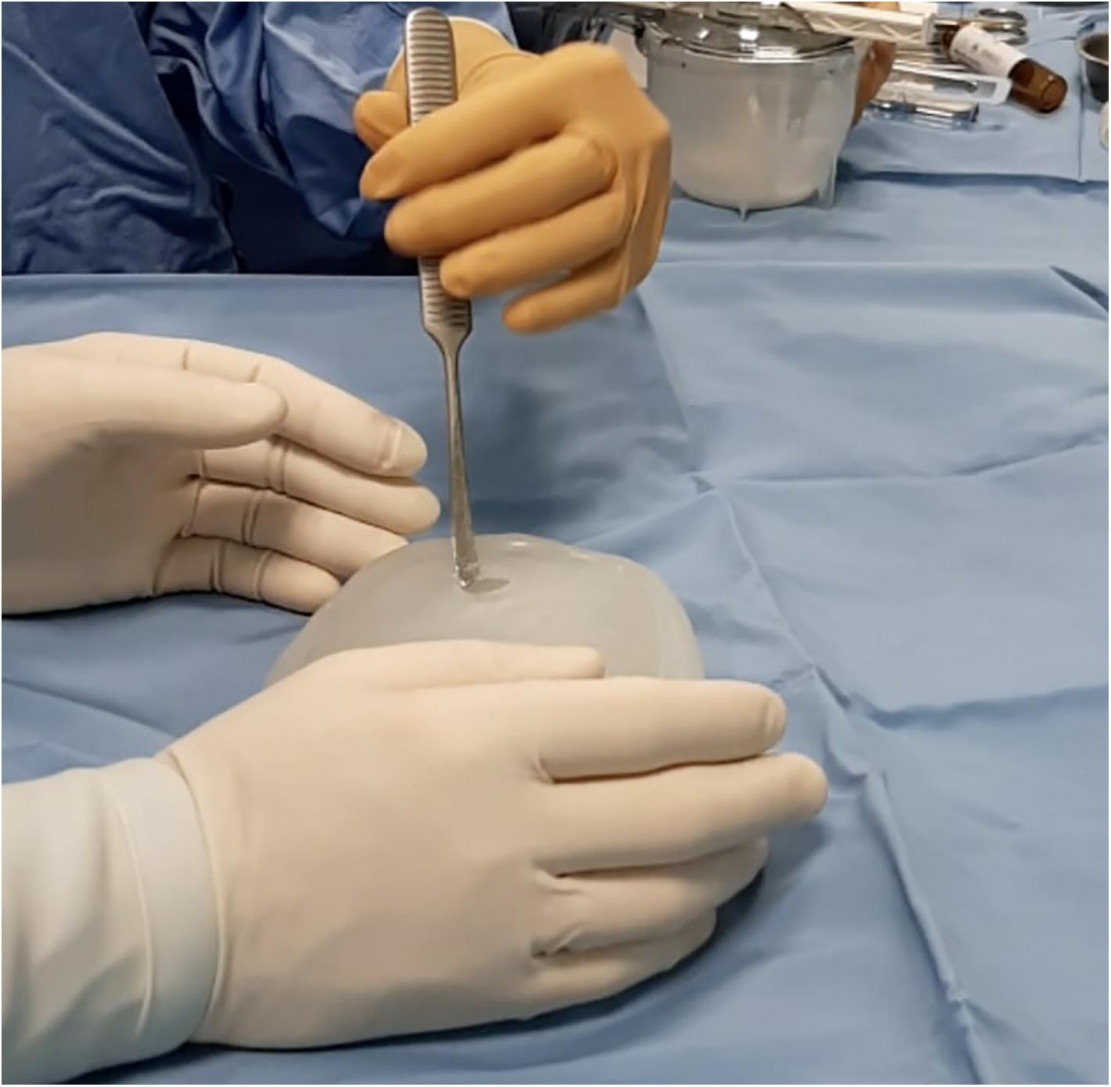
The silicone moulds is unpacked and assembled. From the central hole the inferior part is lowered with a dissector to pour the liquid Polymethyl methacrylate

The silicone mould was unpacked and assembled. From the central hole the inferior part was lowered with a dissector and the liquid PMMA (cranioplastic © by Codman) (Figure 1 and Video 2) was slowly poured into the mould. Creation of air bubbles was prevented by keeping down the inferior part of the mould and slowly raising it once the liquid PMMA distributed all over the inner surface (see Supporting Information S1, Video 2). This valve‐mechanism allows the air to come out with the PMMA in excess.

**FIGURE 2:**
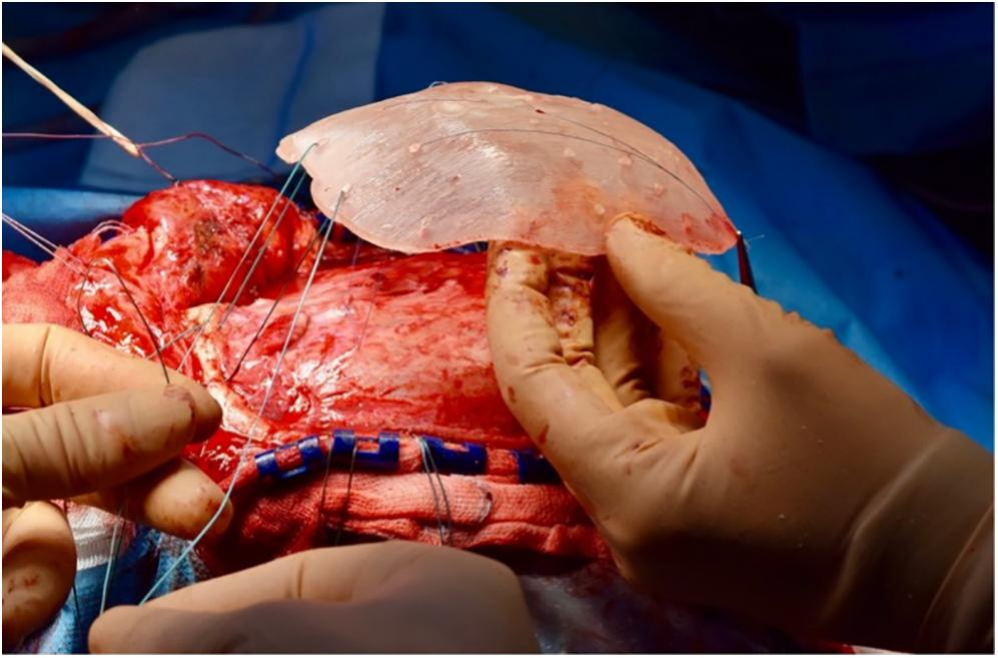
The Polymethyl methacrylate cranioplasty is being placed on the skull defect and attached with mersilene wires

The PMMA implant was left in the silicone mould since it was completely solidified after the exothermic reaction. The solidified cranioplasty was furtherly easily removed from the mould thanks to its elastic properties (see Supporting Information S1, Video 3). We removed small imperfections using a drill and created small holes for dural suspension and osteosynthesis (Figure 2). Finally, the cranioplasty was anchored using mersilene wires.

**FIGURE 3:**
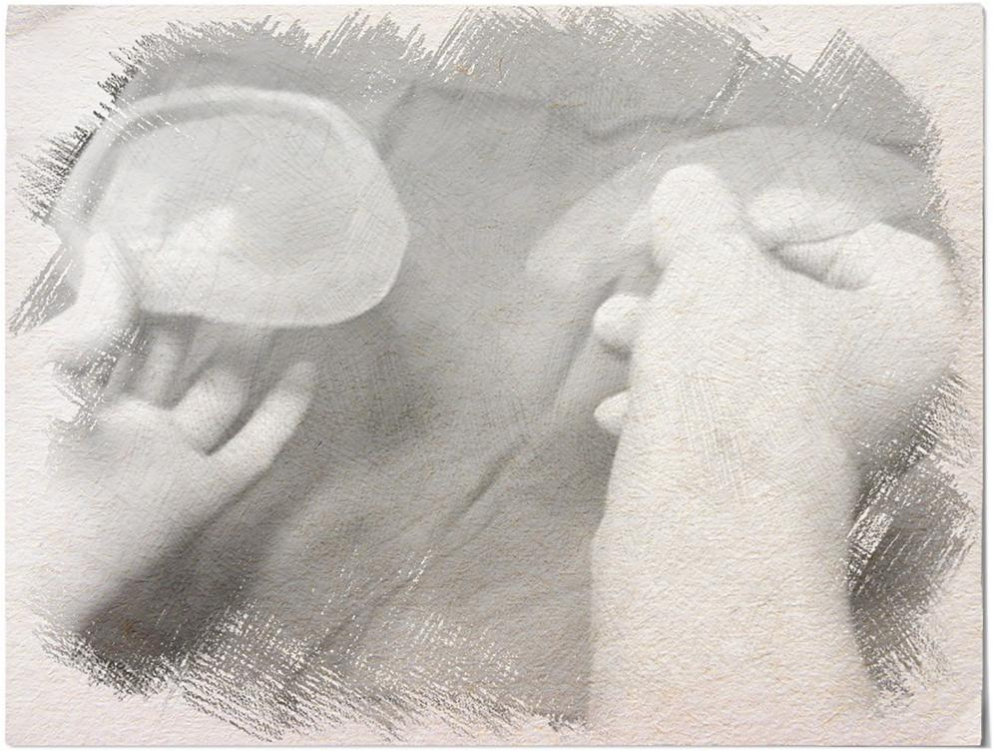
Drawing showing mould system

Figure 3 shows schematic drawings of the whole procedure.

### 2.4 Cost evaluation

We considered the man‐hours required to design the mould and the materials for the mould and cranioplasty.

## 3 RESULTS

We implanted seven patients with the created cranioplasties.

**FIGURE 4:**
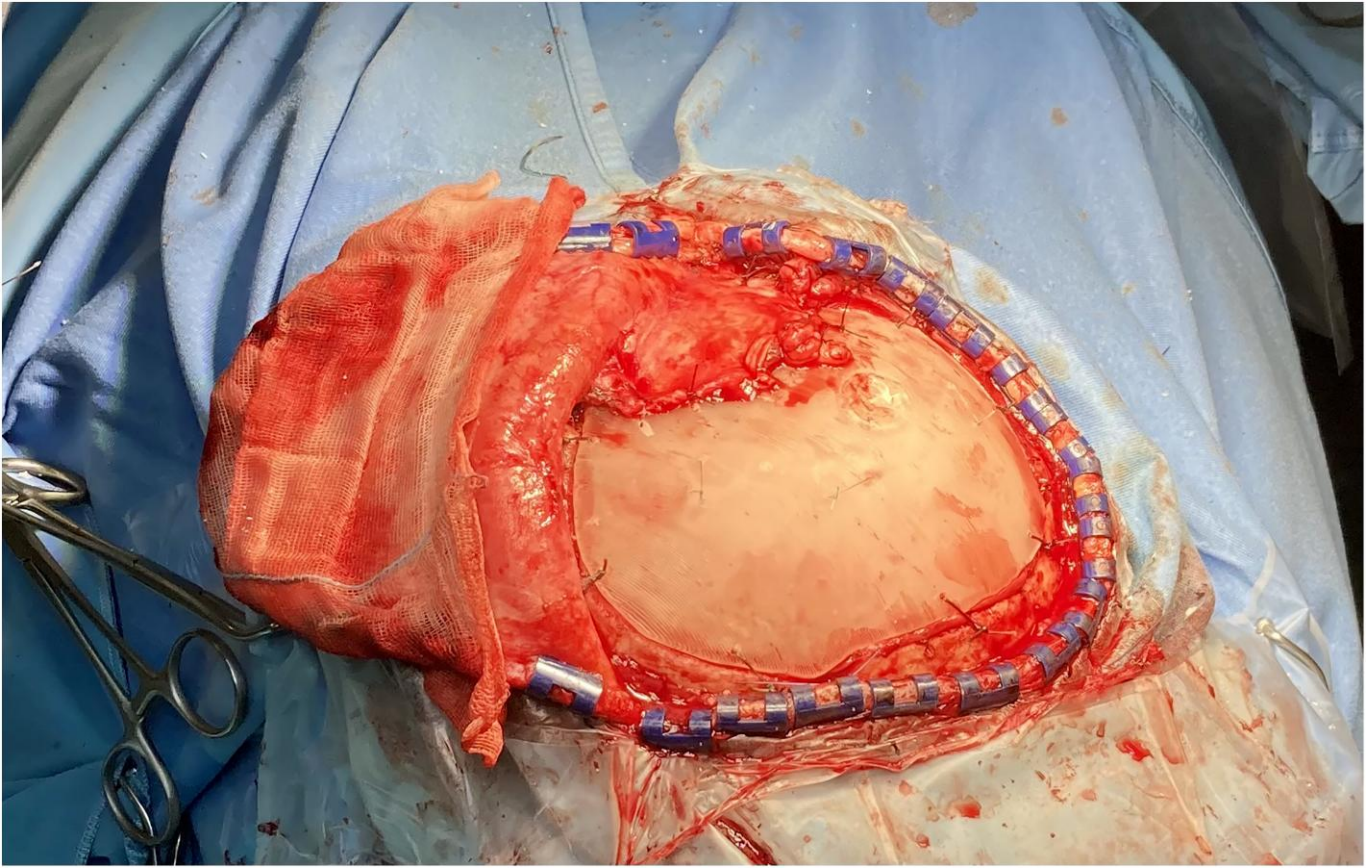
The Polymethyl methacrylate (PMMA) cranioplasty has been placed correctly fitting the defect and temporal muscle attached to guarantee a good cosmetic result

**FIGURE 5:**
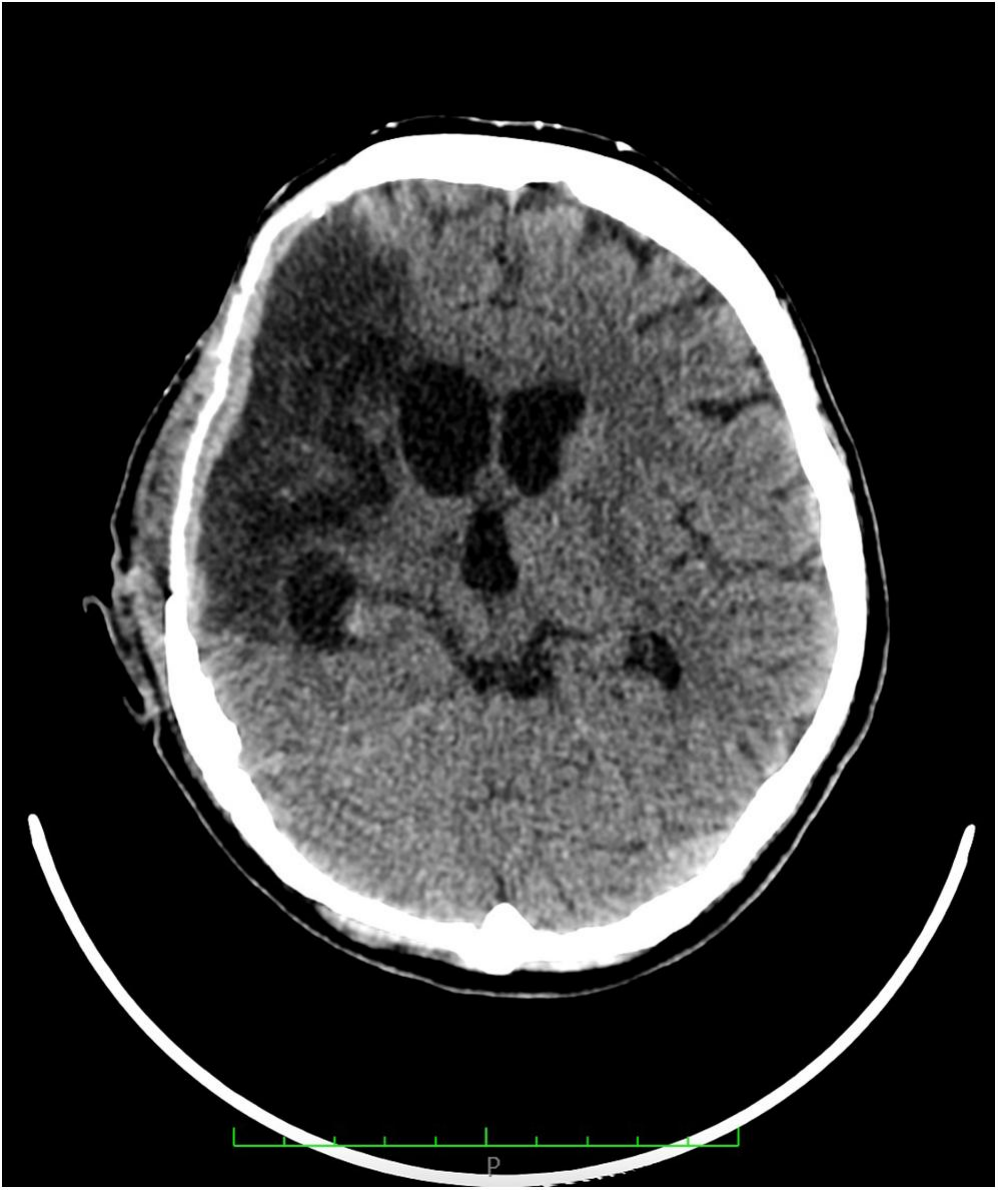
Post‐operative head CT scan showing the correct placement of the cranioplasty

The mean time of surgery was 80 min and all the cranioplasties perfectly matched the defect (Figure 4). Post operative head CT scan showed a good cosmetic result with no complications (Figure 5).

Mean follow‐up was 6 months. No long‐term complications occurred.

Mean cost of the entire procedure was between 2500 and 2700 euros. For specific costs, please see Table 1.

**TABLE 1:** Costs details

Item	
Specialised technician (€/h)	42
Raw material sylicon (€/g)	1.20
Electricity (€/kWh)	0.2181
Raw material per model (g)	200
Depreciation for model	25%
PMMA (€/box)	450

## 4 DISCUSSION

We showed an easy and fast procedure to create and implant a patient‐specific cranioplasty.

Our technique resulted accurate and practical to reach a good cosmetic result.

The accuracy of the template‐moulding is comparable to the directly 3D printed originals.^8^

Several techniques and materials have been reported to produce the 3D printed moulds: biocompatible polyamide,^9^ polylactic acid,^8^ polylactic acid with wax elimination technique,^3^ polycarbonate^10^ or silicone.^11^

We preferred silicone for the following reasons:

1) it can be printed in different types of consistency, and we choose the one which better combines the support of the shape and the simplicity of the PMMA cranioplasty extraction from the mould;
2) it presents an affordable cost;
3) it is autoclavable;
4) it does not stick to the PMMA cranioplasty;
5) it is easily available and tested for medical procedures; Other materials require an adjuvant non‐stick layer such as vaseline^9^ or silicone.^8^

Differently from other authors, we also preferred to pour the PMMA directly in the assembled silicon mould from the central hole. We believe using the assembled mould would reduce the border imperfections you can have performing the procedure in two steps. Moreover, rigid moulds are more difficult to detach from the cranioplasty with a higher risk of damage for the prosthesis.

A recent paper by Dabadi et al.^12^ describes a similar procedure to produce a customised cranial prosthesis. Differently from these authors, we obtain a directly printed 3D silicone mould without previously producing a prosthesis model. In addition, we don't autoclave the PMMA prosthesis but the silicon mould.

Usually, custom made cranioplasty cost is between 4000 and 17,000 euros.^9^

The entire cost of our procedure, including the man‐hours required to design the mould, was 2500–2700 euros. An additional advantage of this technique is the possibility to re‐use the mould in case of infections or cranioplasty damages, sustaining only the cost of the PMMA.

### 4.1 Limitations

We collected a low number of patients.

One of the major limitations is the need of a multidisciplinary team (surgeons, radiologist and engineers) in order to complete the process. In many hospitals, non‐health care professionals such as engineers or 3D printing lab are not readily available. Indeed, hospitals in low‐income countries could not afford to have a residential multidisciplinary team. Such a problem could be overcome by implementing all the project phases (transformation of DICOM files to .stl files and setting of printer parameters) with an automatic software. This software could be released for a very low price (such as 100 euros/year) and be easily accessible online. In this way, the price of the multidisciplinary team could be shot down and the neurosurgeon him/herself could upload the DICOM files, download the .stl file with the appropriate printer settings, and automatically print the mould.

Moreover, when pouring the PMMA in the mould, attention must be taken in order to avoid air bubbles inclusion in the cranioplasty. Indeed, air bubbles can increase the risk of rupture and make diagnostic procedures, such ultrasounds, difficult to perform.

Another possible risk is an increase of infection rate, due to a two steps process (PMMA poured into the mould) instead of using a patient specific sterilised prosthesis. However, we did not experience any infection in the follow‐up period.

## 5 CONCLUSIONS

Several techniques exploiting 3D printing are nowadays available for producing patient‐specific cranioplasties.

The technique we proposed using 3D printed patient specific silicone moulds and PMMA resulted to be effective and affordable ensuring a good cosmetic result.

A multidisciplinary team is necessary; however, we trust modern hospitals are increasingly prone to include non‐health care professional in their teams.

## CONFLICT OF INTEREST

No conflict of interest has been declared by the authors.

## AUTHORS CONTRIBUTIONS

Paolo Zamboni: review and editing (equal). Alba Scerrati: Conceptualisation (lead); writing – original draft (lead); formal analysis (lead); writing – review and editing (equal). Andrea Lombardo: Software (lead); Francesco Travaglini: writing – review and editing (equal). Pasquale De Bonis: Methodology (lead); writing – review and editing (equal). Michele A. Cavallo: Conceptualisation (supporting); Writing – original draft (supporting); Clarissa Ann Elisabeth Gelmi: Writing – review and editing (equal).

## Supporting information

Supporting Information S1

Supporting Information S2

Supporting Information S3

Supporting Information S4

## Data Availability

The data that support the findings of this study are available on request from the corresponding author. The data are not publicly available due to privacy or ethical restrictions.
