# Supplementary material for: Patient specific Polymethyl methacrylate customised cranioplasty using 3D printed silicone moulds: Technical note
Source: Int J Med Robot. 2021 Nov 24;18(2):e2353. doi: 10.1002/rcs.2353 (PMC9285906; doi:10.1002/rcs.2353)
Supplement: Supplementary file 1 — Supporting Information S1 [file RCS-18-0-s002.docx]

**Video Legends**

Video 1. assembly of the two pieces of the silicone mold.

Video 2. Pouring of the PMMA in the silicone mold.

Video 3. After complete solidification of the PMMA the cranioplasty is extracted from the silicone mold.
